# Editorial: Future of cosmetic chemistry: advanced product assessment and chemometrics-assisted evaluation

**DOI:** 10.3389/fchem.2025.1568706

**Published:** 2025-02-13

**Authors:** Vahid Hamedpour, Riccardo Leardi, Jeroen Jansen

**Affiliations:** ^1^ Croda Japan KK, Tokyo, Japan; ^2^ Department of Pharmacy, University of Genova, Genova, Italy; ^3^ Institute of Molecules and Materials, Radboud University, Nijmegen, Netherlands

**Keywords:** modern cosmetic chemistry, chemometrics, efficacy assessment, innovative instrumentation development, novel sample separation

## Introduction

The cosmetics industry is a dynamic and rapidly evolving field, deeply rooted in innovation and scientific advancement. In recent years, it has witnessed a significant shift toward sustainability and consumer-focused formulations, reflecting a growing commitment to safety, environmental responsibility, and high-performance products.

Advances in analytical techniques, including Artificial Intelligence (AI), lab automation, and plant-based innovations, have revolutionized the development and evaluation of cosmetic products. These technologies enable the creation of allergen-free, eco-friendly formulations that meet the increasing demands of informed consumers. Despite these advancements, bridging the gap between consumer needs and effective product solutions remains a key challenge.

This Research Topic was created to introduce cutting-edge approaches for assessing cosmetic products throughout their lifecycle. Focus areas include sustainable ingredient analysis, allergen detection, green chemistry-based analytical methods, design of experiments (DoE) for optimization, advanced imaging technologies, and AI-driven techniques. Emerging platforms like lab-on-a-chip devices and biosensors also offer promising solutions for real-time assessments.

## Highlights of contributions to the Research Topic

The first contribution to this Research Topic presents advanced statistical analyses for cosmetic product developments (Ping). The study tackles the challenge of predicting consumer preferences based on early sensory evaluations, addressing the limitations of traditional blind-use tests (BUTs), which are both time-consuming and costly. By introducing an innovative approach using Bayesian bootstrapping, the paper enhances the predictive accuracy of expert-conducted sensory testing. This method significantly improves the alignment between early evaluations and subsequent BUT outcomes, offering a streamlined and efficient framework for the cosmetic product development process.

The second contribution introduces an innovative approach to objectively assess makeup foundation coverage (Blaksley et al.). Traditional sensory evaluations, often inconsistent, are replaced with hyperspectral imaging to analyze detailed spectral data from facial skin before and after application. Key parameters, homogeneity factor (α_HF_) and spectral shift factor (β_SF_), quantify coverage consistency and color changes, respectively. The study demonstrates strong correlations between these metrics and sensory rankings, offering a reproducible, quantitative method to evaluate makeup coverage.

The next contribution to this Research Topic reported by Ruscinc et al. evaluates the safety and efficacy of four polyphenols (chlorogenic acid, apigenin, kaempferol, and naringenin) commonly used in cosmetic formulations. This study employs a combination of *in-vitro* and *in-vivo* methods, including the hen’s egg test on chorioallantoic membrane (HET-CAM) for irritation potential, high-performance liquid chromatography–thiobarbituric acid reactive substances–*ex vivo* stratum corneum (HPLC-TBARS-EVSC) for assessing antioxidant activity, and laser Doppler flowmetry to measure anti-inflammatory effects. Findings indicate that while all tested polyphenols are non-irritating, only naringenin significantly reduces lipid peroxidation in the stratum corneum, suggesting superior antioxidant properties.

The fourth contribution by Mancuso et al., reviews the application of reflectance spectroscopy as a non-invasive analytical technique for assessing skin responses to topical formulations. By analyzing reflected light across various wavelengths, this method quantifies skin parameters such as erythema index and melanin content, enabling the monitoring of physiological changes induced by cosmetic or pharmaceutical products. This study discusses the principles of reflectance spectroscopy, its implementation in dermatological research, and its advantages over traditional invasive methods like biopsies.

## Summary

The rise in consumer awareness has enhanced the demand for innovative, safe, and high-performance cosmetic products, driving advancements in evaluation methodologies. We hope this Research Topic serves as a valuable resource, to inspire continued innovation and offer insights into the tools and strategies that will shape the future of cosmetic science.

